# A case of concurrent exacerbation of manifestations and asthma after the initiation of nemolizumab treatment for atopic dermatitis

**DOI:** 10.1016/j.jdcr.2025.03.026

**Published:** 2025-04-10

**Authors:** Michie Katsuta, Ayaka Osawa, Yozo Ishiuji, Satomi Fujii, Masahiro Kanbe, Akihiko Asahina, Yoshimasa Nobeyama

**Affiliations:** Department of Dermatology, Jikei University School of Medicine, Minato-ku, Tokyo, Japan

**Keywords:** asthma, atopic dermatitis, IL-31, itch, nemolizumab, Th2

## Introduction

Nemolizumab is a monoclonal antibody targeting interleukin (IL)-31 receptor α. The drug is available for the treatment of moderate to severe atopic dermatitis (AD) by its subcutaneous administration dosed with 60 mg every 4 weeks. In particular, the agent is known to be highly effective for itch in AD. However, a high incidence rate of cutaneous adverse events of the treatment has been reported.^1^ Here, we experienced a case that both cutaneous manifestations and asthma were exacerbated after the initiation of nemolizumab treatment for AD. Here, we report this case to provide the insight for making a decision in the choice of therapeutic options for the patients with both AD and asthma.

## Case report

**Fig 1:**
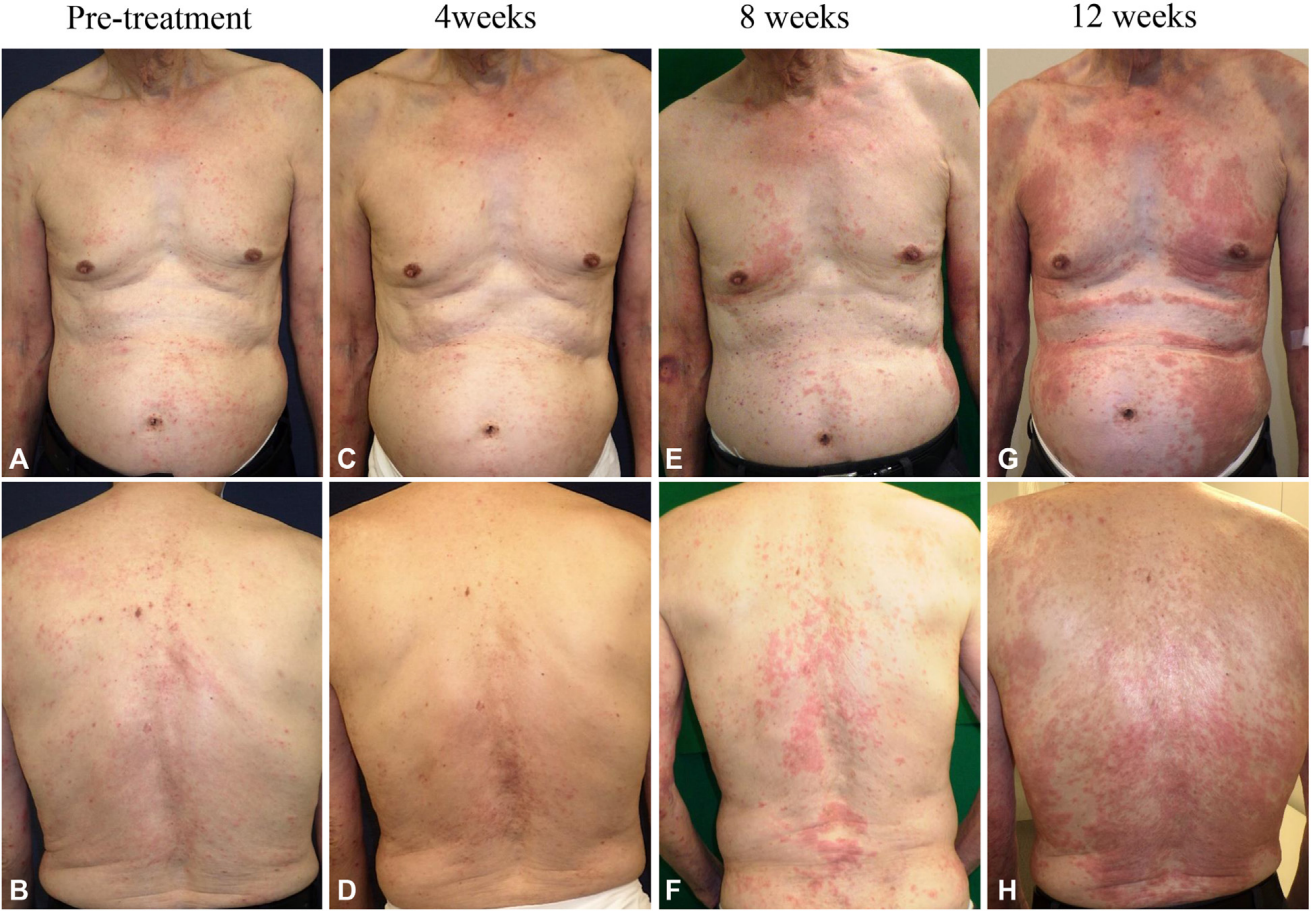
**A, B,** Clinical presentation at the start of nemolizumab treatment; **C, D,** at 4 weeks; **E, F,** at 8 weeks; and **G, H,** at 12 weeks.

**Fig 2:**
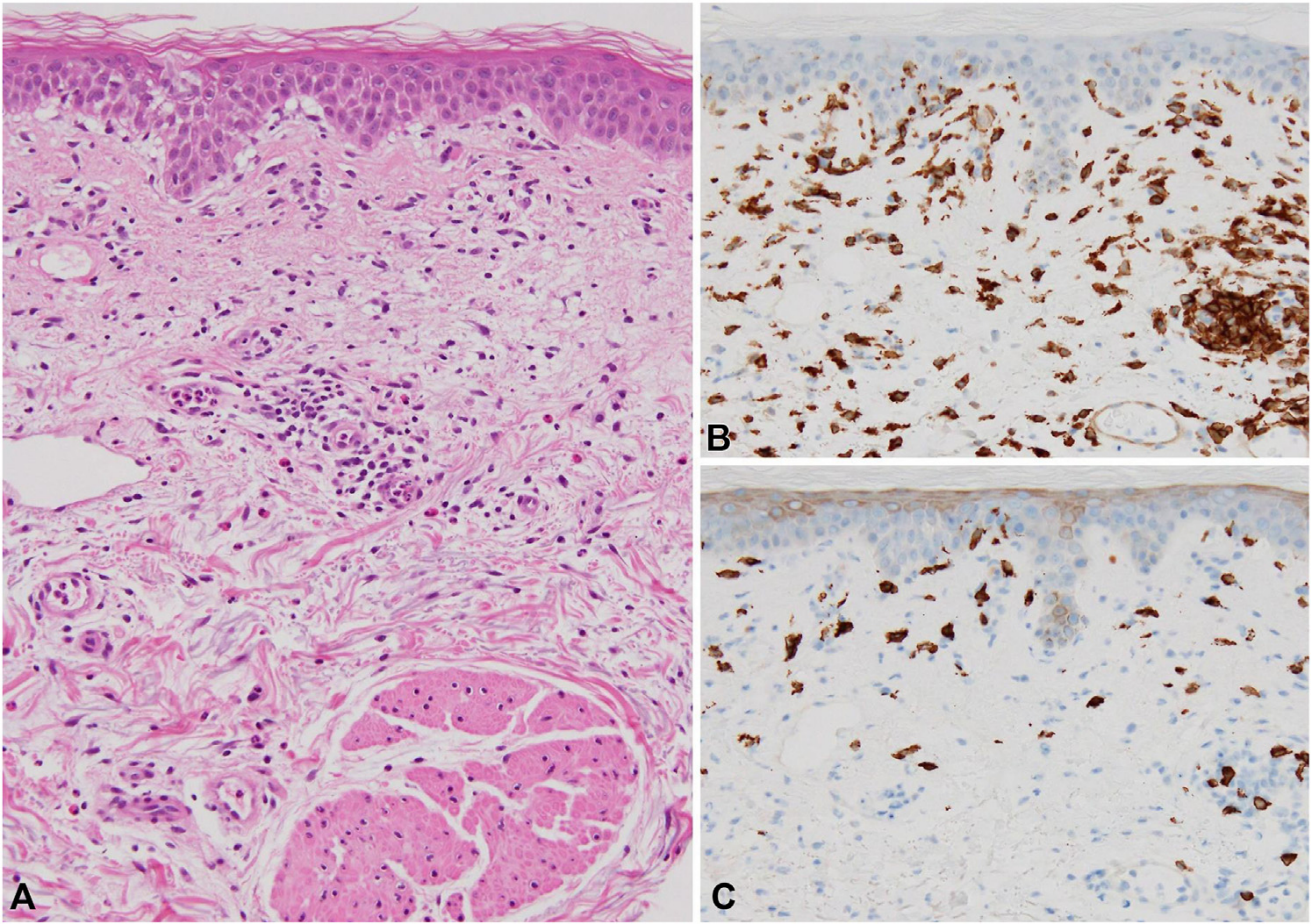
**A,** Histopathologic findings showing liquefactive degeneration of the dermis and infiltration of eosinophils; **B,** immunohistochemistry CD4^+^ cells; **C,** immunohistochemistry CD8^+^ cells.

An 81-year-old Japanese man was referred to our hospital with the chief complaint of severe itch associated with AD that met Hanifin and Rajka’s diagnostic criteria^2^ and was poorly controlled despite treatment with class II topical corticosteroids. The patient had asthma, which had been well-controlled with no attacks for at least 5 years. The disease was kept into well-controlled status with 25 points by the Asthma Control Test, a self-administered questionnaire to subjectively evaluate asthma control status with the scores ranging from 5 to 25 (5-19 indicates very poorly controlled, 20-24 poorly controlled, and 25 controlled).^3^ Before our treatment, the numeric rating scale score for itch was 8 (range, 0-10) and Eczema Area and Severity Index was 12 (range, 0-72) (Fig 1, *A, B*). We started to administer nemolizumab 60 mg subcutaneously for the treatment of AD. The second week after the initiation of the administration, erythema and papules, which were previously undetected, appeared on the trunk, and then the second dose of nemolizumab 60 mg was administered (Fig 1, *C, D*). However, the third dose of nemolizumab was discontinued because the manifestations were more progressive (Fig 1, *E, F*). Concurrently, the patient began experiencing asthma attacks at night requiring inhaled corticosteroids for the first time in 5 years, and his Asthma Control Test score decreased to 18. At the 12th week, erythematous macules and papules newly appeared further (Fig 1, *G, H*). Skin biopsy was performed from newly emerging erythematous macules and papules on the trunk. Histopathologic examination demonstrated a liquefactive degeneration, spongiosis, and dermal infiltration of inflammatory cells, including lymphocytes and eosinophils (Fig 2, *A*). Immunohistochemical findings demonstrated a higher number of CD4^+^ cells than CD8^+^ cells in the epidermis and papillary dermis (Fig 2, *B, C*). Blood examinations revealed an increased number of eosinophils, and elevated levels of serum thymus and activation-regulated chemokine and immunoglobulin (Ig) E (eosinophils increased from 1065 to 1159 /mm^3^, thymus and activation-regulated chemokine from 358 to 1084 pg/mL, and IgE from 269 to 905 IU/mL). The serum anti-BP 180 antibody level was within the normal range (<9 U/mL). Based on these findings, we considered that both the exacerbation of the cutaneous manifestations and the asthma attacks were associated with nemolizumab treatment. Therefore, his asthma attack was treated with low dose of systemic corticosteroid (betamethasone 0.02 mg/kg/d) and inhaled corticosteroid, resulting in improvement of both asthma attacks and the cutaneous manifestations. Consequently, systemic therapies such as biologics and Janus kinase inhibitors were not required for AD. We reduced dose of systemic corticosteroid (betamethasone 0.0067 mg/kg/d) after his asthma had been well-controlled (Asthma Control Test 25). No asthma attacks occurred 2 years after stopping nemolizumab treatment. He has continued to be treated with topical steroids and difamilast ointment (Eczema Area and Severity Index 6.3, numeric rating scale 2).

## Discussion

Our patient experienced deterioration of the cutaneous manifestations accompanied by an increase in peripheral blood eosinophil count and serum thymus and activation-regulated chemokine levels, although the patient was released from itch after nemolizumab administration. Inhibition of IL-31 by nemolizumab activates IL-13 associated with a T helper 2 (Th2)-predominant condition, potentially causing cutaneous adverse events.^4^

There are some reports on the role of IL-31 regarding asthma. IL-31 was reported to be an indicator of asthma severity with a proinflammatory function positively correlated with Th2 cytokines in peripheral blood.^5^ In contrast, inflammation appeared in association with increased levels of serum IgE, IL-4, and IL-13 in transgenic mice where the IL-31 or IL-31 receptor α(IL-31RA) signaling pathway is impaired, indicating IL-31 plays an anti-inflammatory role.^6^ To resolve conflict, Huang et al^6^ has been suggested biphasic functions of IL-31 or IL-31RA axis. The axis may act as a proinflammatory signaling system associated with secretion of chemokines to recruit inflammatory cells in the early phase of allergic asthma. On the other hand, the axis may act an anti-inflammatory signaling system limiting Th2 cytokine responses in the late phase.^6^

In a phase 2B study, Silverberg et al^7^ suggested that asthma after initiation of nemolizumab is less severity of respiratory symptoms and occurs in patients with a history of asthma. However, there have been no reports of asthma treatment-emergent adverse events in phase 3 study.^8^ The results of reported asthma adverse events differed between phase 2B study and phase 3 study: in a phase 2B study, all asthma attacks occurring up to week 24 were recorded, whereas in phase 3 study, only treatment-emergent adverse events with a prevalence of ≥5% after the first dose of nemolizumab were reported. This may be one of the reasons why the description of asthma attacks as an adverse reaction differed between the phase 2B study and the phase 3 study.

Based on the above findings, we considered that the suppression of IL-31 by nemolizumab in the present case resulted in asthma attacks and exacerbation of cutaneous manifestation. The present study for the first time suggests that nemolizumab potentially induces activation of well-controlled asthma through the suppression of IL-31, which has a critical role for asthma inactivation.

## Conflicts of interest

Drs Katsuta, Nobeyama, and Asahina received research funding and honorariums from Maruho as speakers. Dr Ishiuji received an honorarium as a speaker from Maruho, Sanofi, Abbvie, Pfizer, Eli Lilly, and Otsuka. The other authors have no conflicts of interest to declare.
